# American Medical Association

**Published:** 1867-04

**Authors:** W. B. Atkinson

**Affiliations:** 215 Spruce St., Philadelphia


### *American Medical Association.*

The Eighteenth Annual Meeting of the *American Medical Association* will be held in Cincinnati, on Tuesday, May 7th, 1867, at 11 o'clock A. M.

The following Committees are expected to report:

On Quarantine, Dr. Wilson Jewell, Pa., chairman.

On Ligature of Subclavian Artery, Dr. Willard Parker, N. Y., chairman.

On Progression of Medical Science, Dr. Jerome C. Smith, N. Y., chairman.

On the Comparative Value of Life in City and Country, Dr. Edward Jarvis, Mass., chairman.

On Drainage and Sewerage of Cities, &c., Dr. Wilson Jewell, Pa., chairman.

On the Use of Plaster of Paris in Surgery, Dr. James L. Little, N. Y., chairman.

On Prize Essays, Dr. F. Donaldson, Md., chairman.

On Medical Education, Dr. S. D. Gross, Pa., chairman.

On Medical Literature, Dr. A. C. Post, N. Y., chairman.

On Instruction in Medical Colleges, Dr. Nathan S. Davis, Ill., chairman.

On Rank of Medical Men in the Army, Dr. D. H. Storer, Mass., chairman.

On Rank of Medical Men in the Navy, Dr. W. M. Wood, U. S. N., chairman.

On Insanity, Dr. Isaac Ray, R. I., chairman.

On American Medical Necrology, Dr. C. C. Cox, Md., chairman.

On the Causes of Epidemics, Dr. Thomas Antisell., D. C., chairman.

On Compulsory Vaccination, Dr. A. N. Bell, N. Y., chairman.

On Leakage of Gas-Pipes, Dr. J. C. Draper, N. Y., chairman.

On Alcohol and its Relations to Man, Dr. J. R. W. Dunbar, Md., chairman.

On the Various Surgical Operations for the Relief of Defective Vision, Dr. M. A. Pallen, Mo., chairman.

On Local Anæsthesia, Dr. E. Krackowitzer, N. Q., chairman.

On the Influence upon Vision of the Abnormal Conditions of the Muscu'ar Apparatus of the Eye, Dr. H. D. Noyes, N. Y., chairman.

On the Comparative Merits of the Different Operations for the Extraction of Vesicle Calculi, Dr. B. J. Raphael, N. Y., chairman.

On the Therapeutics of Inhalation, Dr. J. Solis Cohen, Pa., chairman.

On the Deleterious Articles used in Dentistry, Dr. Augustus Mason, Mass., chairman.

On Medical Ethics, Dr. Worthington Hooker, Conn., chairman.

On the Climatology and Epidemics of Maine, Dr. J. C. Weston.

Of New Hampshire, Dr. P. A. Stackpole.
Vermont, Dr. Henry Janes.
Massachusetts, Dr. Alfred C. Garrett.
Rhode Island, Dr. C. W. Parsons.
Connecticut, Dr. B. H. Catlin.
New York, Dr. E. M. Chapman.
New Jersey, Dr. Ezra M. Hunt.
Pennsylvania, Dr. D. F. Condie.
Delaware, Dr. — Wood.
Maryland, Dr. O. S. Mahon.
Georgia, Dr. Juriah Harriss.
Missouri, Dr. Geo. Engelman.

Alabama, Dr. R. Miller.
Texas, Dr. Greensville Dowell.
Illinois, Dr. R. C. Hamil.
Indiana, Dr. J. F. Hibberd.
District of Columbia, Dr. T. Antisell.
Iowa, Dr. J. W. H. Baker.
Michigan, Dr. Abm. Sager.
Ohio, Dr. J. W. Russell.

Secretaries of all Medical Organizations are requested to forward lists of their Delegates as soon as elected, to the Permanent Secretary.

W. B. ATKINSON,
215 *Spruce St.*, *Philadelphia.*

